# The Millennial Generation and “The Lecture”

**DOI:** 10.1111/j.1553-2712.2011.01215.x

**Published:** 2011-11

**Authors:** Danielle Hart, Scott Joing, John Burton

**Affiliations:** Department of Emergency Medicine Hennepin County Medical CenterMinneapolis, MN

In this 30-minute talk, we will briefly discuss the characteristics of the Millennial Generation and explore in depth how these characteristics relate to their ability, or inability, to learn productively in a traditional lecture-style format. This discussion will encompass both a review of the current literature and survey results from the current emergency medicine residents at Hennepin County Medical Center regarding techniques they find effective and engaging during “the lecture” or other large group educational sessions. The goal of this talk is to expand or refine the armamentarium of tools to use when designing your large-group educational sessions, to maximize the learning and retention of information for your students.

## Supporting Information

The following supporting information is available in the online version of this paper:

**Video Supplement S1.** The Millennial Generation & “The Lecture.”
